# Deposition of a SiO_2_ Shell of Variable Thickness and Chemical Composition to Carbonyl Iron: Synthesis and Microwave Measurements

**DOI:** 10.3390/s21144624

**Published:** 2021-07-06

**Authors:** Arthur V. Dolmatov, Sergey S. Maklakov, Polina A. Zezyulina, Alexey V. Osipov, Dmitry A. Petrov, Andrey S. Naboko, Viktor I. Polozov, Sergey A. Maklakov, Sergey N. Starostenko, Andrey N. Lagarkov

**Affiliations:** 1 Institute for Theoretical and Applied Electromagnetics RAS, Izhorskaya St. 13, 125412 Moscow, Russia; dolmatov.av@phystech.edu (A.V.D.); zez-p@yandex.ru (P.A.Z.); avosipov@mail.ru (A.V.O.); dpetrov-itae@yandex.ru (D.A.P.); nas.webwork@gmail.com (A.S.N.); viktor.polozov@phystech.edu (V.I.P.); sergeymaklakov@yandex.ru (S.A.M.); snstar@mail.ru (S.N.S.); maklakov@itae.ru (A.N.L.); 2 Moscow Institute of Physics and Technology, National Research University, 9 Institutskiy per., 141700 Dolgoprudny, Russia

**Keywords:** protective coating, soft magnetic powder, microwave permittivity, core–shell particles

## Abstract

Protective SiO_2_ coating deposited to iron microparticles is highly demanded both for the chemical and magnetic performance of the latter. Hydrolysis of tetraethoxysilane is the crucial method for SiO_2_ deposition from a solution. The capabilities of this technique have not been thoroughly studied yet. Here, two factors were tested to affect the chemical composition and the thickness of the SiO_2_ shell. It was found that an increase in the hydrolysis reaction time thickened the SiO_2_ shell from 100 to 200 nm. Moreover, a decrease in the acidity of the reaction mixture not only thickened the shell but also varied the chemical composition from SiO_3.0_ to SiO_8.6_. The thickness and composition of the dielectric layer were studied by scanning electron microscopy and energy-dispersive X-ray analysis. Microwave permeability and permittivity of the SiO_2_-coated iron particles mixed with a paraffin wax matrix were measured by the coaxial line technique. An increase in thickness of the silica layer decreased the real quasi-static permittivity. The changes observed were shown to agree with the Maxwell Garnett effective medium theory. The new method developed to fine-tune the chemical properties of the protective SiO_2_ shell may be helpful for new magnetic biosensor designs as it allows for biocompatibility adjustment.

## 1. Introduction

Iron powders are widely applied in power transformers, inductors, sensors [1], electromagnetic compatibility solutions [2,3], and materials designed to decrease electromagnetic pollution [4,5]. Commonly used is the carbonyl iron with spherical particles of 2 [5,6]–10 [4] micron in the mean diameter. Carbonyl iron possesses remarkable magnetic properties, although chemical stability and electromagnetic performance are still to be improved when embedded into composite materials. Surface modification of iron particles with a chemically inert non-conductive coating may solve these tasks. Inorganic and polymer coatings suit well: MnO_2_, BaTiO_3_, carbon, PMMA, polyaniline [4], parylene C [7], ZnO, Fe_3_O_4_ [8], etc. In addition, the SiO_2_ is the most commonly used among others. Silica shell provides iron with oxidation resistance in the air [4,9] and corrosion resistance [6]. Modifying iron with these non-conductive shells prevents electric contact between particles and combines magnetic and dielectric losses in one material [8]. This may be used to fine-tune the electromagnetic properties of the latter [6,9,10].

Liquid-phase hydrolysis of silica precursors is frequently used due to its simplicity and effectiveness, although other techniques, even mechanical milling [11], also work. Recently publications on synthesizing iron powders coated with silica shells show the high importance of these studies. The primary requirement for the silica shell is uniformity. While simple tetraethyl orthosilicate (TEOS) hydrolysis in ammonia solution deposits uniformly coating to nano- and micro-particles of iron, modification of large, 200-μm-particles requires additional surface activation. Surface active agents were shown to successfully increase the uniformity of the SiO_2_ coating (see references within [12]). Mechanical milling also improves the adhesion of the silica coating to coarse iron powders [12]. Another technique to increase the uniformity of the SiO_2_ is the use of L-lysine instead of ammonia solution [4].

Despite all these studies, it is notable that the inherent properties of the silica shell, including chemical composition and dielectric constant, are rarely examined. However, it is known that the hydrolysis of organosilanes gives significantly different products depending on the acidity of the reaction mixture, which, in turn, is governed by ammonia concentration [13,14,15,16]. For example, two different final-stage thermal treatments may be applied for the Fe@SiO_2_ drying. One is simply to keep the powder at 50–60 °C for a certain period [5,6,17]. The other is to anneal in an inert (N_2_, [8]) or reducing atmosphere (H_2_, [1]) at 500–800 °C for several hours. The latter decreases the oxygen content of the silica down to SiO_1.5_ [8]. This is due to the volatilization of water that occurs at 100 °C and further thermal aging of the SiO_2_ [8].

From pure silica gel studies, it is known that the real chemical composition affects the dielectric constant of the “SiO_2_”. Hydroxyl groups are those elements in the structure of the silica that are dealt with by polarization. There are at least two types of hydroxyls within the silica structure: intraglobular and surface hydroxyls (although the former may be further divided into subclasses, and some “free” hydroxyls are also distinguishable). The surface or “perturbed” hydroxyls are deemed to cause polarization via a constrained rotation; that is, a rotation from one hydrogen-bonded position to another [18]. It was also shown that annealing at a temperature up to 1000 °C gradually decreases the concentration of hydroxyl groups, increases density, decreases surface area, and decreases the dielectric constant of the silica. The maximum decrease is from 2.2 to 1.8, almost 20% of the initial value (see Table 2 from ref. [18]). The density of the silica was shown to influence the permittivity of the silica even in the GHz range [19].

These changes may not only be used to tune electromagnetic performance but to develop a desired biocompatibility of the Fe@SiO_2_-based media. Particle size and surface charge of the silica are the key parameters in biocompatibility studies [20]. A method to optimize the surface charge of the carbonyl iron particles coated with the silica shell makes the powder a promising material for biomedical applications. The powder may be applied both in pure form or incorporated into some silicone matrix to form a magnetorheological elastomer [21]. The latter serves as a magnetic-field magnetostriction sensor (more precisely, a dual-mode magnetism/pressure sensor), which sensing performance is closely related to the matrix–polymer interactions that, in turn, are governed by surface modification of iron particles [21,22].

Here, the SiO_2_ shell was deposited onto carbonyl iron of 3 μm mean diameter through hydrolysis of TEOS in water–ethanol solution. Duration of the hydrolysis reaction and ammonia concentration was studied to affect the thickness and chemical composition of the shell. The products were dried in the air under 60 °C for 6 h. Chemical composition was measured by EDX using scanning electron microscopy. Microwave measurements in the 0.5–15 GHz range were used to evaluate the permeability of the composite material comprised of the Fe@SiO_2_ particles and paraffin wax matrix. The measured real permeability of the composite was shown to be in accordance with what was calculated following the Maxwell Garnett formula [23,24,25].

## 2. Materials and Methods

The deposition of the SiO_2_ shell onto particles of carbonyl iron (CI) was carried out in a one-stage modified Stöber process. Pure carbonyl iron powder (≥97.0 mass.% Fe), tetraethyl orthosilicate (CAS 78-10-4, Aldrich №86578), and ammonia solution (25%, reagent grade) were used. First, 2 g of the metal powder was immersed in ethanol in a round-bottom 100-mL flask with a reflux condenser. The tetraethyl orthosilicate was added and ultrasonicated for 40 min with a power of 250 W and a frequency of 40 kHz. Then, ammonia was added to the mixture, and this was assigned as the start of the reaction.

Two different experiments were conducted. The first experiment studied the influence of the duration of the hydrolysis reaction on the properties of the product. The duration was 0.5, 1, 2, and 4 h. The ratio of volumes of TEOS and ammonia solution added to the reaction mixture, here and after [TEOS]/[NH_3_·H_2_O], was constant at 1. Another experiment studied the influence of concentrations of reagents on the properties of the product. The [TEOS]/[NH_3_·H_2_O] ratio was 0.75, 1, 1.5, and 4.5. The duration of the synthesis was 2 h in this experiment.

Whatever the process was, the product was separated by magnet-assisted decantation, rinsed multiple times in ethanol until the transparency of the liquid, and dried in air for 6 h at 60 °C.

Particle size and SiO_2_ thickness were measured by scanning electron microscopy using the Zeiss Evo 50 VP microscope (Zeiss AG, Germany). Fifteen individual particles were studied and averaged to evaluate the thickness of the shell. Energy-dispersive X-ray (EDX) analysis (Si:O) was examined using the “Oxford instruments” platform (Oxford instruments, UK). A sample area of 15 × 15 μm was studied.

**Figure 1:**
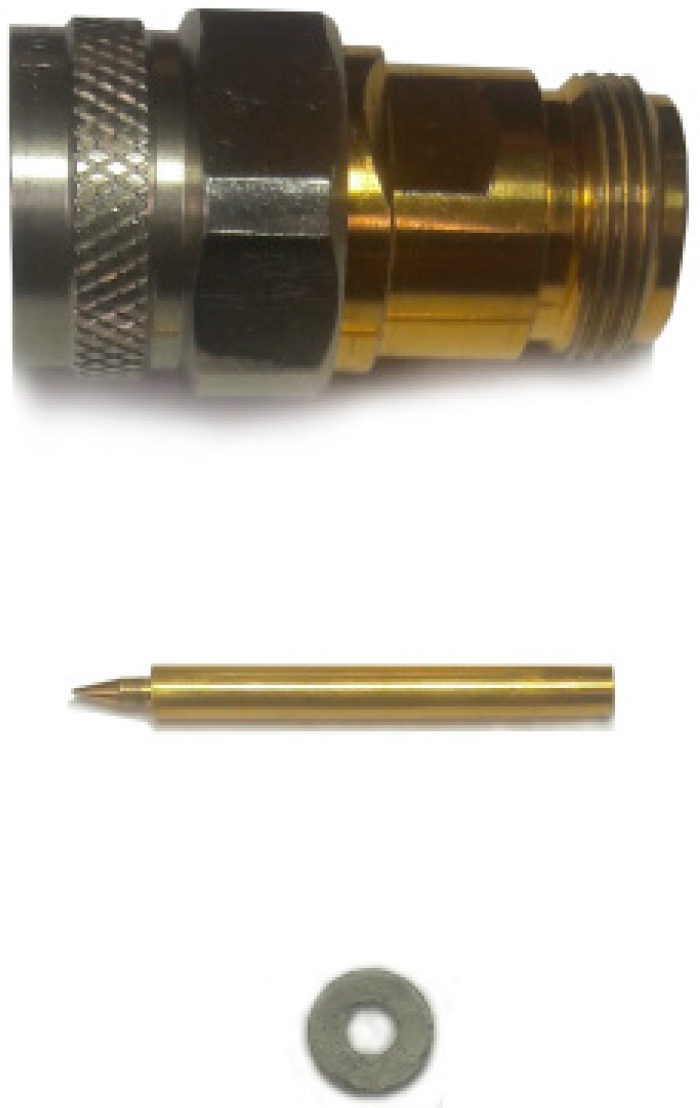
Coaxial transmission line (on top) with a central conductor (in the middle) and a composite sample (at the bottom) for microwave measurements.

The composites for microwave measurements were made of Fe@SiO_2_ particles mixed with molten paraffin wax with constant stirring during cooling [6]. The volume fraction of the Fe@SiO_2_ powder was estimated at 35 vol.%. The volume fraction was calculated as follows. Initially, 30 mg of the filler was mixed with 100 mg of the wax. The shell thickness was set at 150 nm, since the real size distribution of iron particles and the presence of pure silica particles interferes with more precise calculations. The tabular density of iron was 7.8 g/cm^3^, and the density for amorphous silica was 2.2 g/cm^3^. The samples were formed in the torus and placed inside a standard 7/3 mm coaxial transmission line (Figure 1). Additionally, the sample inside the line was pressed slightly from both ends to force the composite material to fill the cross-section of the line and reduce possible air gaps between the sample and the metallic surface of the line. 

S-parameters of the composite samples placed in the airline were measured in the frequency range of 0.5 to 15 GHz with a vector network analyzer (VNA). Ports at the end of feeding coaxial cables were calibrated with standard TRL calibration procedure [26] with planes of phase reference at the ends of the measurement transmission line. The complex microwave permeability and permittivity were determined with the standard Nicolson–Ross–Weir (NRW) [27,28] method.

The quasi-static permittivity of the composites was determined at a frequency of 500 MHz. The frequency dependence of complex permittivity was fitted with the Havriliak–Negami empirical formula [29] to minimize possible errors. The measured data were fitted in the frequency range of 0.5 to 10 GHz to minimize low-frequency errors due to poor sensitivity of the microwave measurements at frequencies lower than 0.5 GHz. At frequencies higher than 10 GHz, the half-wavelength resonance on the sample thickness starts to affect, and NRW solution becomes inherently unstable.

Higher-order modes can also emerge on the sample boundary with typical resonance-like behavior of the calculated microwave permittivity and permeability [30,31]. Such resonance behavior cannot be attributed to the material properties and should be carefully considered. In our case, the sample length was chosen to force the possible emergence of higher-order modes beyond the frequency range of interest. However, for some samples, the effect of higher-order modes resonance on the sample length can be seen at frequencies higher than 10 GHz. The manifestation of this effect depends on the sample homogeneity and quality of the geometric shape and cannot be easily controlled.

The lower bound of the frequency region under study was chosen due to the following considerations. In the frequency range from 10 MHz to 500 MHz, TRL calibration performance starts to degrade due to the small phase difference in the transmission line calibration standard and direct-thru connection of the measurement ports. In addition, samples with small electric length, i.e., small permittivity, permeability, and geometric length, have low reflection coefficient in this frequency region (i.e., low contrast); thus, the NRW method performance also degrades. 

## 3. Results and Discussion

### 3.1. Structure and Morphology

**Figure 2:**
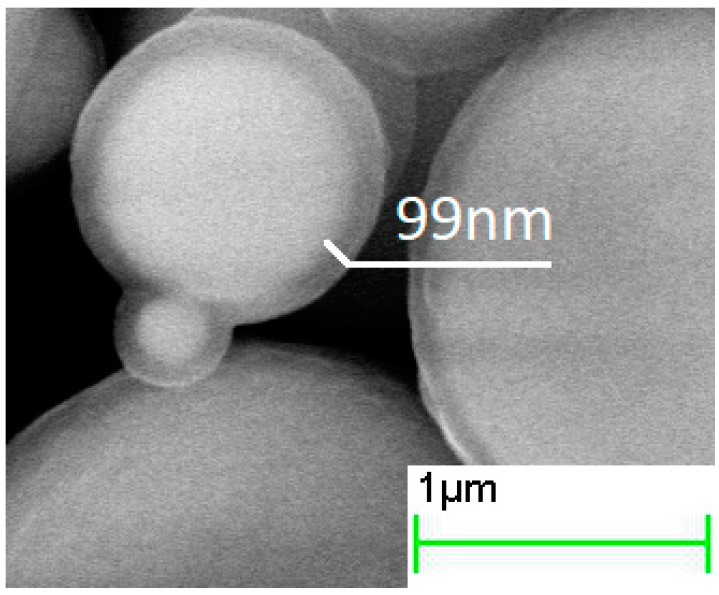
SEM images of core–shell particles obtained in the processes with 0.5 h hydrolysis duration.

**Figure 3:**
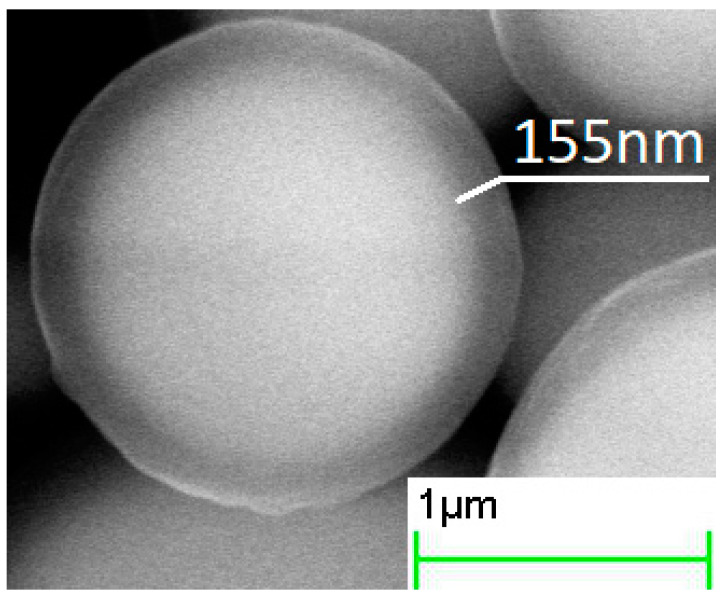
SEM images of core–shell particles obtained in the processes with 2 h hydrolysis duration.

**Figure 4:**
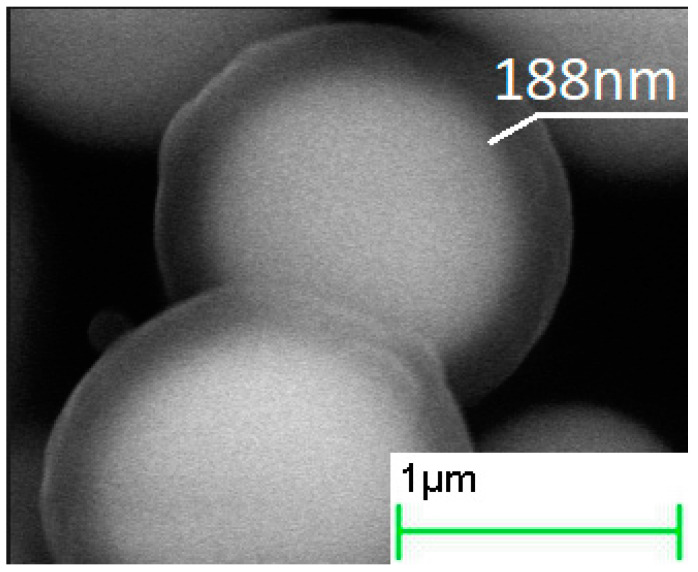
SEM images of core–shell particles obtained in the processes with 4 h hydrolysis duration.

**Figure 5:**
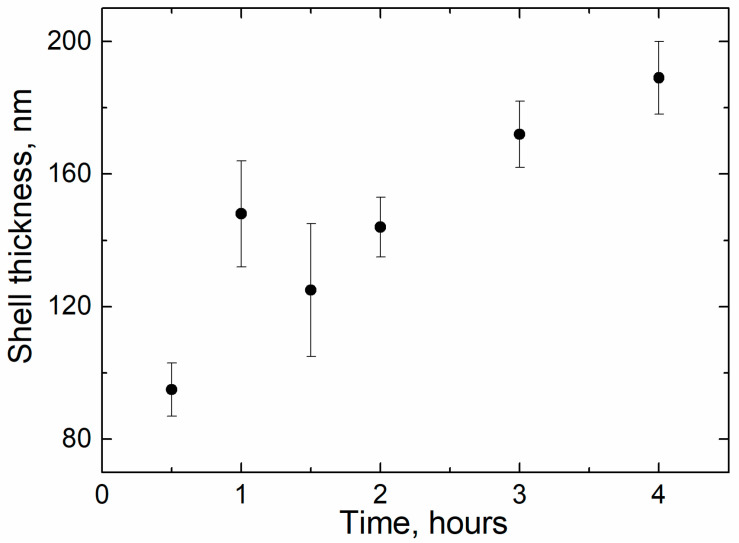
SiO_2_ shell thickness vs. hydrolysis duration, “time”.

The increase in the duration of the deposition process from 0.5 to 4 h increased the thickness of the silica layer (Figure 2, Figure 3 and Figure 4, Table 1). The minimum thickness obtained was 90 nm, and the maximum was 190 nm (Figure 5). Further prolongation of the process did not increase thickness. The distribution of the shell thickness in a sample narrowed with the reaction time (Table 1). Uniformity and final particle size of the silica are known to be dealt with by colloid interaction potentials. The reaction rate here is size-dependent and is governed by the competition between nucleation and aggregation [32]. Growth of the shell thicker than 200 nm was probably inhibited by the colloidal surface state of the product. The shell thickness evaluated from one-hour-deposited samples differed slightly from the monotonous trend from other data. This was probably a random error caused by a wide distribution of the shell thicknesses that were deposited with a duration of 1–1.5 h. No evidence was observed that the thickness of the shell might decrease with the reaction time in this synthesis.

According to EDX analysis, the changes in the duration of the reaction did not affect the stoichiometry of the “SiO_x_”. The atomic ratio Si:O remained at SiO_3.3_. Excess of oxygen is in the form of hydrate water and surface hydroxyls, the real composition being SiO_2_·*n*H_2_O. An increase in atomic Si:Fe ratio was also observed, and it also showed an increase in the silica content within the samples subjected to the longer deposition process.

**Figure 6:**
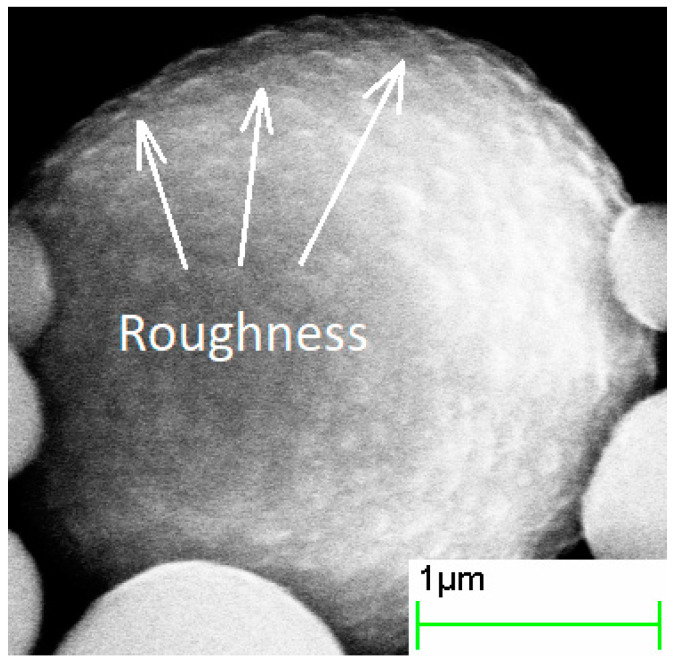
SEM images of core–shell particles obtained in the process with [TEOS]/[NH_3_·H_2_O] = 4.5 ammonia concentration.

**Figure 7:**
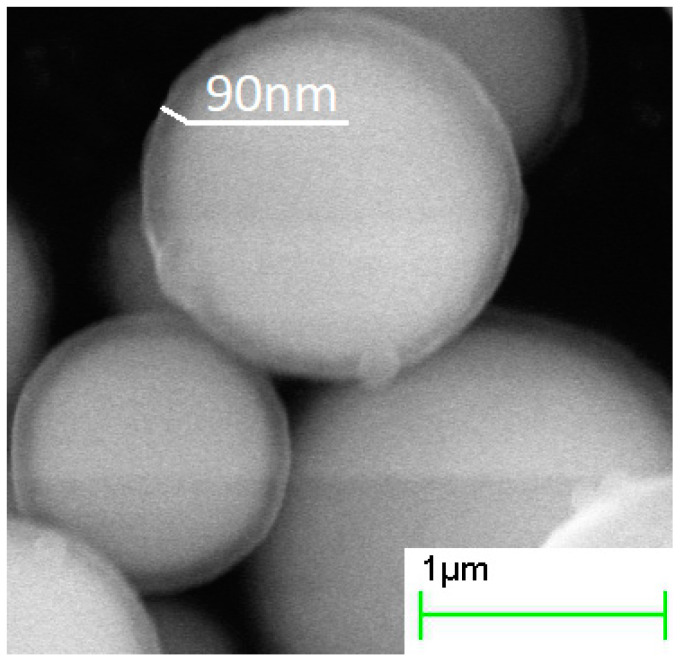
SEM images of core–shell particles obtained in the process with [TEOS]/[NH_3_·H_2_O] = 1.5 ammonia concentration.

**Figure 8:**
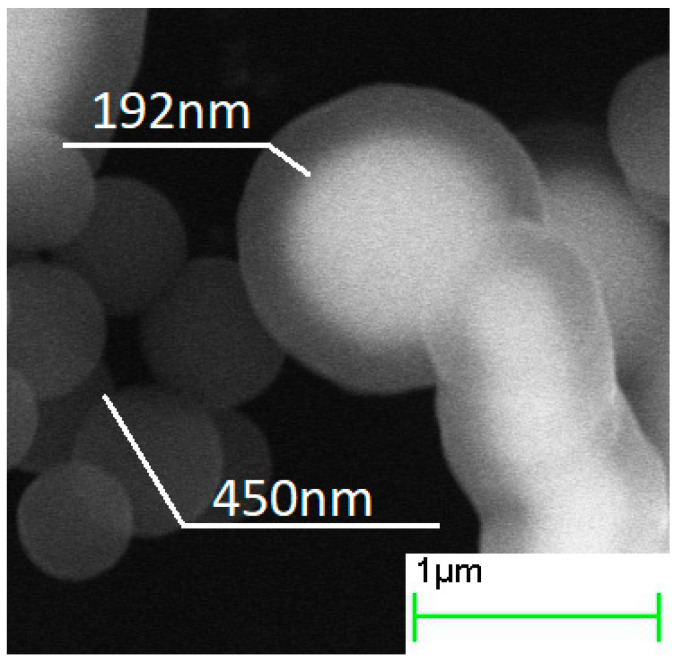
SEM images of core–shell particles obtained in the process with [TEOS]/[NH_3_·H_2_O] = 0.75 ammonia concentration.

**Figure 9:**
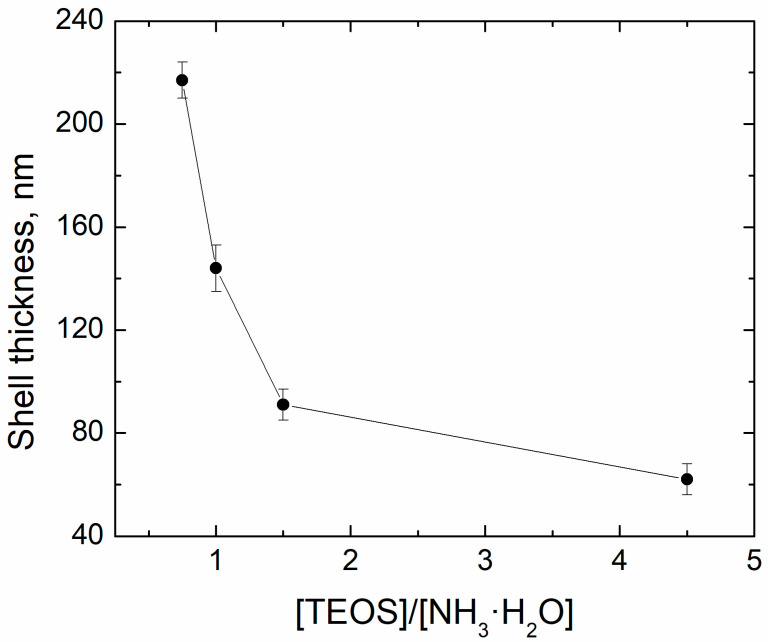
The thickness of the shell of the Fe@SiO_2_ filler varied through *ammonia concentration* thickness of the shell vs. [TEOS]/[NH_3_·H_2_O] ratio.

Ammonia concentration was found to influence both the thickness and chemical composition of the shell (Figure 6, Figure 7 and Figure 8, Table 2). Minimal thickness was estimated at ~60 nm, and it was deposited under low ammonia concentration. The thickest shell was deposited in an excess of ammonia, and it was ~220 nm (Figure 9). An increase in the [NH_3_·H_2_O] concentration resulted in a shell significantly enriched with oxygen, as much as SiO_8.6_. An excess of ammonia also resulted in an enlarged fraction of individual SiO_2_ nanoparticles with a mean size that was twice as high as the estimated thickness of the shell. The surface of particles obtained under the excess of ammonia was smooth, while those deposited under [TEOS]/[NH_3_·H_2_O] = 4.5 showed surface roughness of approximately 80 nm (Figure 6). These results are in agreement with the data on how ammonia influences the growth of the silica gel [33].

It is interesting to note that the distribution of the thickness of the silica shell was narrower in those cases when the duration of hydrolysis was longer than 2 h (compare the confidence interval given in Table 1 when the duration time was 0.5, 1, and 1.5 h, and the rest of the confidence intervals provided in Table 1 and Table 2). It may be assumed that during the first 1.5 h, the growth rate of the silica shell differs for iron particles of different sizes and the resulting thickness levels during the time interval between 1.5 and 2 h of the hydrolysis reaction. The deviation of the mean shell thickness coincides sufficiently with the deviations calculated in [32], where the monodisperse silica was grown and studied.

### 3.2. Dielectric Permittivity: Theoretical Approach

In order to evaluate the dependence of the real part of the static relative permittivity of the core–shell particles on the thickness of the shell, the Maxwell Garnett effective medium theory was used. The theory allows calculating effective permittivity of the media comprised of two materials, one of which is a matrix and another one is inclusion. The Maxwell Garnett theory is valid in the case of a small concentration of the inclusion [34]. However, in a quasi-static regime, it is applicable for any inclusion concentration [35]. Effective permittivity of the composites in the quasi-static field was calculated as:

(1) $$\begin{array}{c} \varepsilon_{eff}=\varepsilon_{h}+3f\varepsilon_{h}\frac{\varepsilon_{i}-\varepsilon_{h}}{\varepsilon_{i}+2\varepsilon_{h}-f\left(\varepsilon_{i}-\varepsilon_{h}\right)}\; \end{array}$$

where $\varepsilon_{h}$ and $\varepsilon_{i}$ were the relative permittivity of the matrix and inclusion, respectively, and *f* was a volume fraction of the inclusion. For the present Fe@SiO_2_ composite $\varepsilon_{h}=\varepsilon_{SiO_{2}}=3,9$ [36], $\varepsilon_{i}=\varepsilon_{Fe}=\infty$ and $f=\frac{R_{Fe}^{3}}{{\left(R_{Fe}+t\right)}^{3}}$, where $R_{Fe}$ = 1500 nm was iron particle radius [6] and t was the thickness of the dielectric shell. The thickness varied from 30 to 210 nm. Taking $\varepsilon_{i}$ as ∞ in (1) results in the following formula for $\varepsilon_{eff}$

(2) $$\begin{array}{c} \varepsilon_{eff}=\varepsilon_{h}+3f\varepsilon_{h}\frac{1}{1-f}\; \end{array}$$

**Figure 10:**
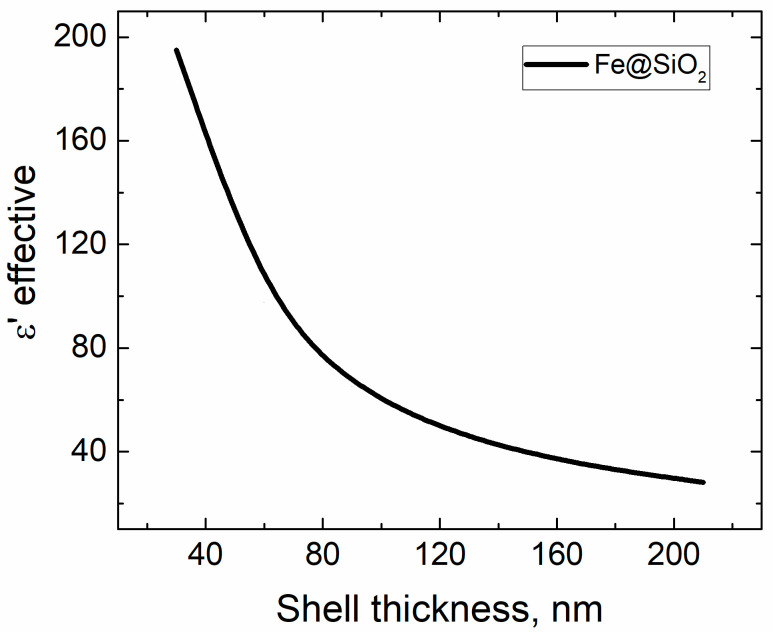
Calculated effective permittivity vs. SiO_2_ shell thickness of the Fe@SiO_2_ “composite” Fe@SiO_2_–paraffin wax real composite.

The theoretical model showed a decrease in permittivity by 86%, with an increase in the thickness of the shell from 30 to 210 nm (Figure 10). The particle size distribution of the initial iron powder can be found in Figure 3 from [6]. Another calculation technique may be applied, which is first calculating the effective permeability of the paraffin and silicon shell, and then using that result as the environmental permittivity for the iron [37]. However, if the model applied in the manuscript may be derived rigorously with account for inclusion and shell shapes, another model is semi-empirical.

**Figure 11:**
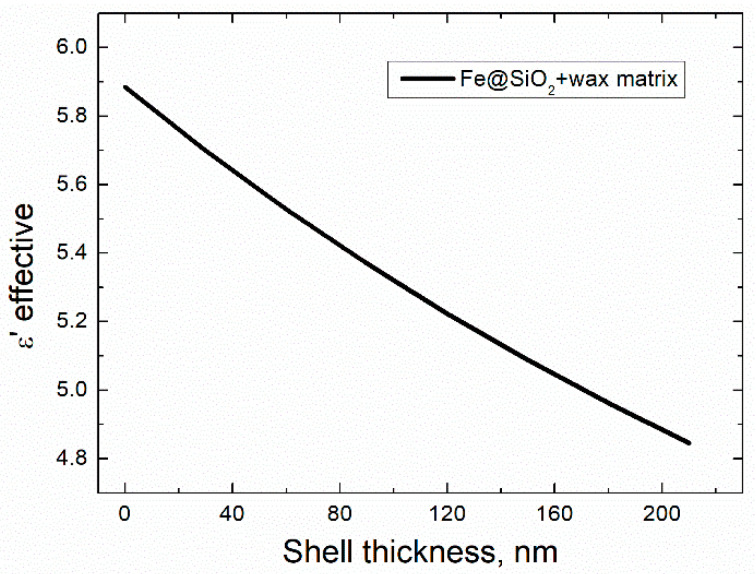
Calculated effective permittivity vs. SiO_2_ shell thickness of the Fe@SiO_2_–paraffin wax real composite.

Measurements of the permittivity demanded blending of the Fe@SiO_2_ particles into paraffin matrix. Consequently, it was necessary to evaluate the effective permittivity of the system composed of core–shell particles and paraffin in order to compare experimental and theoretical results. The permittivity was calculated using the Maxwell Garnett theory as well [38]. For the Fe@SiO_2_ + Paraffin medium $\varepsilon_{h}=\varepsilon_{Paraffin}=2.25$ [38], $\varepsilon_{i}=\varepsilon_{Fe@SiO_{2}}$, which was calculated previously, and f=0.35. In the presence of the paraffin matrix dramatic decrease in the permittivity was smoothed, allowing fine tuning of the *ε*′. The permittivity dropped by 11%: at shell thickness of 30 nm, $\varepsilon_{Fe@SiO_{2}+Paraffin}=5.69,$ while at 150 nm $\varepsilon_{Fe@SiO_{2}+Paraffin}=5.09$ (Figure 11).

The permittivity of the pure CI in the paraffin medium (shell thickness of 0 nm at Figure 11) was estimated according to (2) with $\varepsilon_{m}=\varepsilon_{Paraffin}=2.25$ and f=0.35.

### 3.3. Frequency Dispersions of Complex Permittivity and Permeability

**Figure 12:**
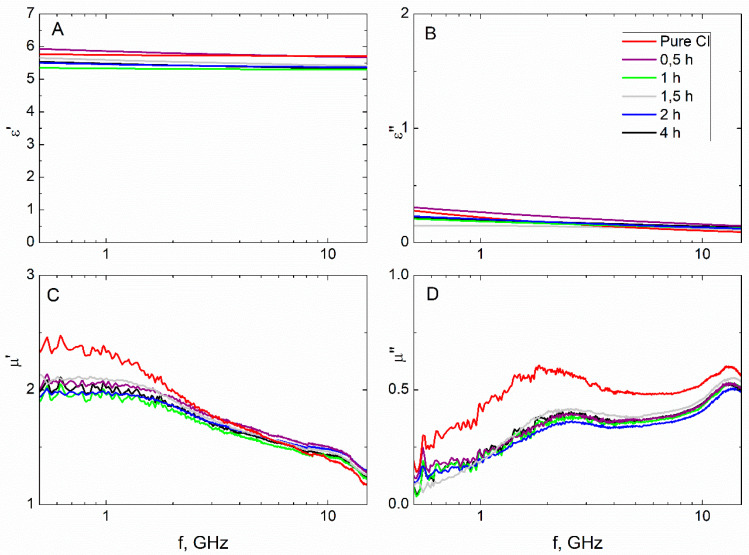
The measured frequency dispersions of complex permittivity, *ε*′ (**A**) + *i·ε*″ (**C**), and permeability, *μ*′ (**B**) + *μ*″ (**D**) for the Fe@SiO_2_–paraffin wax composite. The thickness of the shell of the Fe@SiO_2_ filler varied through *hydrolysis duration*.

**Figure 13:**
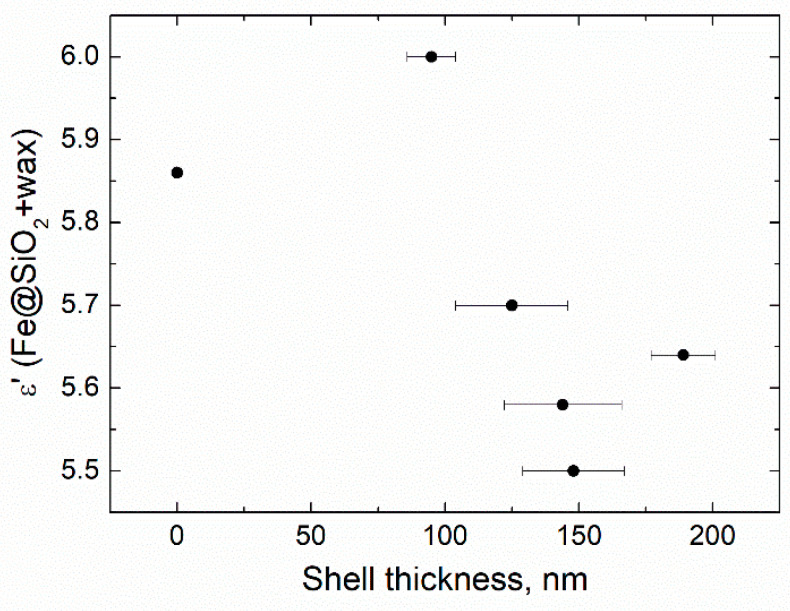
Quasi-static permittivity vs. thickness of the dielectric shell deposited at different durations of hydrolysis.

Analysis of the reaction time variation allows assessing the impact of thickening of the dielectric shell on electromagnetic properties (*ε* and *μ*) of the composite. Samples acquired in more prolonged reactions tended to have a lower real part of permittivity (*ε*′), consistent with a thicker shell (Figure 12A). Values of the *ε*′ in the quasi-static regime demonstrated a good agreement with the theoretical estimation based on the Maxwell Garnett effective medium theory (Figure 13). Frequency dispersions of the *ε*′ of pure CI and the composite obtained in the half-hour reaction were indistinguishable, demonstrating that the shell was not uniform yet in a half-hour experiment. The shell can be considered uniform, starting with 90–100 nm thickness.

An increase in the shell thickness did not influence the imaginary part of the permittivity (*ε*″). The *ε*″ accounts for a loss in the medium. In the analyzed composites, conductive loss in the CI was a primary source of loss since SiO_2_ and paraffin wax are low-loss materials (their dielectric loss tangents are ~0.002 [39] and ~0.007 [40]). Therefore, the absence of the changes in the *ε*″ dispersion demonstrated that the shell growth process does not affect the conductivity of the CI.

Both parts of the complex permeability (*μ*′ and *μ*″) of the composite were lower than in the pure CI that indicates a larger volume fraction of magnetic material in the composite samples. The behavior of the frequency response curves of *μ*′ and *μ*″ was the same for the pure CI and composites that implies that magnetic properties of the CI were unaffected by the shell.

**Figure 14:**
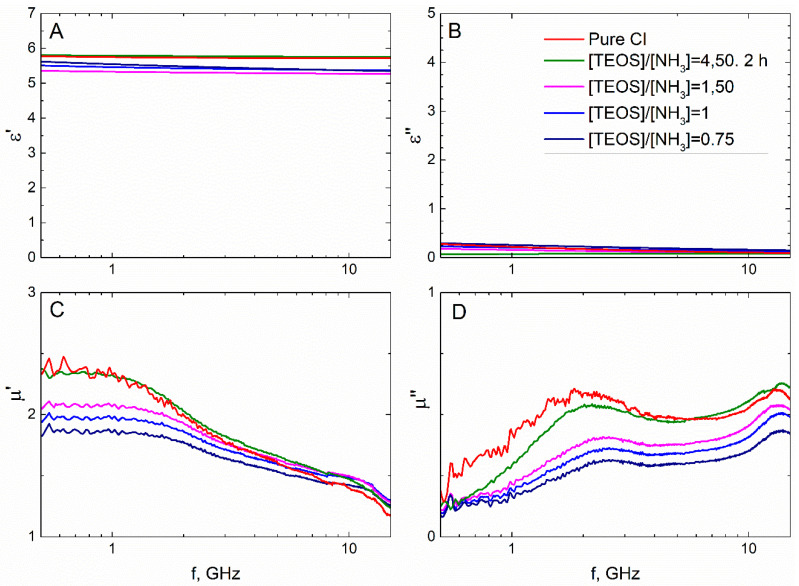
The measured frequency dispersions of complex permittivity, *ε*′ (**A**) + *i·ε*″ (**C**), and permeability, *μ*′ (**B**) + *μ*″ (**D**) for the Fe@SiO_2_–paraffin wax composite.

**Figure 15:**
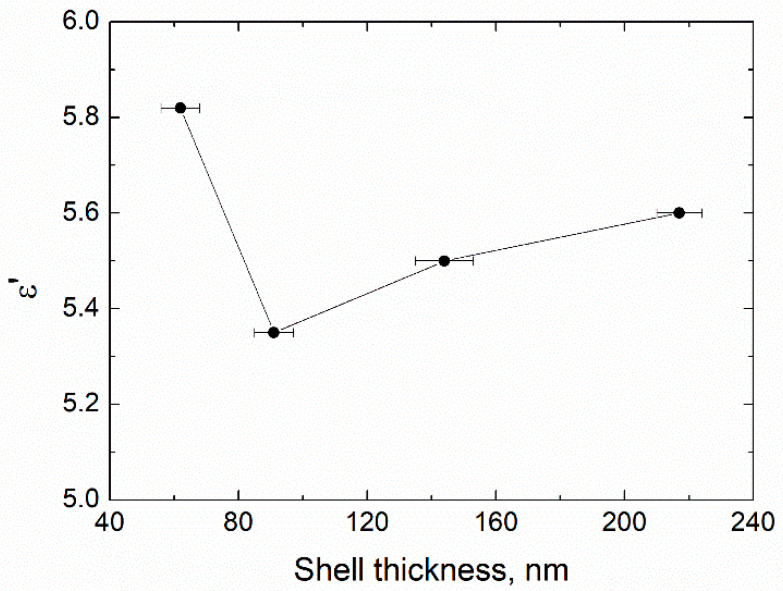
Quasi-static real part of permittivity vs. thickness of the shell that was obtained by varying ammonia concentration.

The measured permittivity showed that a thin coating of 60 nm was not uniform: the *ε*′ value was almost identical to that of pure iron powder-based composite (Figure 14 and Figure 15). The amplitude of permeability also supported this proposition: thin SiO_2_ shells did not significantly decrease this parameter. Further increase in thickness dropped quasi-static *ε*′ value from 5.8 to 5.3, by 9%. This was also according to the Maxwell Garnett calculations, just as in the duration of the hydrolysis experiment. However, the increase in oxygen content within the SiO_x_ composition resulted in a slight increase in the *ε*′. This, in turn, was probably due to an increase in the *ε*′ value of the dielectric shell itself, according to [18].

For the rest of the observations, the *ε*″, *μ*′ and *μ*″ demonstrated the same dependencies on thickness as in the hydrolysis duration experiment (Figure 14). Changes in the chemical composition of the SiO_x_ did not vary the magnetic properties of the composite since SiO_x_ possesses no magnetic order. The *ε*″ did not depend on the shell thickness and composition, showing that SiO_x_ is a low-loss dielectric.

## 4. Conclusions

Hydrolysis of TEOS in the presence of ammonia of different concentrations gave uniform SiO_x_ coating on a surface of carbonyl iron micro-particles when the thickness of the coating was higher than 100 nm. Two techniques were found to increase the thickness of the shell up to approximately 200 nm. One was simply to prolong the duration of the deposition reaction to 4 h. Further prolongation was found to be ineffective for increasing the thickness. After deposition and drying at 60 °C, the shell composition was estimated at SiO_3.3_. The other technique was to change the ammonia concentration in the reaction mixture: an increase in [NH_3_·H_2_O] concentration increased the thickness of the shell when the duration of the deposition was constant. Simultaneously, [NH_3_·H_2_O] was found to influence the composition of the shell. In relative terms, a decrease in [TEOS]/[NH_3_·H_2_O] ratio from 4.5 to 0.75 enriched the silica with oxygen from SiO_3.0_ to SiO_8.6_. Although it can be expected that the difference in chemical composition may vary the dielectric properties of the silica, the difference in the electromagnetic performance of the Fe@SiO_2_ core–shell powders was found to be governed primarily by the thickness of the shell. This was estimated comparing the measured real permittivity values of the Fe@SiO_2_–paraffin wax composites and theoretical values calculated following the Maxwell Garnett formula. The new method to easily fine-tune the chemical composition and thickness of the uniform silica shell deposited to carbonyl iron particles may be instructive for microwave performance and biocompatibility adjustment in a wide range of applications, including magnetic field sensors.

It can be expected, both from experience and the literature data given in the introduction section, that the dependencies reported here will remain the same when the size of the iron core is higher than 3 micrometers, up to at least 200–500 micrometers. With the decrease in the size of iron particles, an effective fraction of the SiO_2_ shell will increase, which will undoubtedly affect the magnetic properties of the product. This effect may be expected to be the most obvious when protecting iron nanoparticles instead of microparticles. However, in general, the size of iron particles is deemed not to affect the mechanisms of the SiO_2_ formation.

## Figures and Tables

**Table 1:** The mean thickness with the confidence interval (*p* = 0.9) of the silica shell, measured from the hydrolysis duration experiment.

Hydrolysis duration, h	0.5	1	1.5	2	3	4
Shell thickness, nm	95 ± 11	148 ± 22	125 ± 23	144 ± 13	172 ± 12	189 ± 14

**Table 2:** The mean thickness with the confidence interval (*p* = 0.9) of the silica shell, measured from the ammonia concentration experiment.

[TEOS]/[NH_3_·H_2_O] ratio	4.5	1.5	1	0.75
Shell thickness, nm	62 ± 9	90 ± 8	146 ± 12	218 ± 10
Si:O atomic ratio	SiO_3.0_	SiO_3.3_	SiO_3.8_	SiO_8.6_

## Data Availability

Data is contained within the article.
